# Adipokines, Vitamin D, and Selected Inflammatory Biomarkers among Parkinson’s Disease Patients with and without Dyskinesia: A Preliminary Examination

**DOI:** 10.3390/metabo14020106

**Published:** 2024-02-05

**Authors:** Jan Milanowski, Jarosław Nuszkiewicz, Beata Lisewska, Paweł Lisewski, Karolina Szewczyk-Golec

**Affiliations:** 1 Student Research Club of Medical Biology and Biochemistry, Department of Medical Biology and Biochemistry, Faculty of Medicine, Ludwik Rydygier Collegium Medicum in Bydgoszcz, Nicolaus Copernicus University in Toruń, 24 Karłowicza St., 85-092 Bydgoszcz, Poland; 2 Department of Medical Biology and Biochemistry, Faculty of Medicine, Ludwik Rydygier Collegium Medicum in Bydgoszcz, Nicolaus Copernicus University in Toruń, 24 Karłowicza St., 85-092 Bydgoszcz, Poland; 3 Medical Center “Neuromed”, 14 Jana Biziela St., 85-163 Bydgoszcz, Poland

**Keywords:** adipokines, dyskinesia, inflammation, neurodegeneration, neuroinflammation, Parkinson’s disease, vitamin D

## Abstract

Parkinson’s disease (PD), a widely recognized neurodegenerative disorder, is characterized by a spectrum of symptoms including motor fluctuations and dyskinesia. Neuroinflammation and dysregulation of adipokines are increasingly implicated in the progression of PD. This preliminary study investigated the levels of inflammatory biomarkers and adipokines, namely interleukin-6 (IL-6), tumor necrosis factor α (TNF-α), C-reactive protein (CRP), visfatin, progranulin, and 25(OH)-vitamin D in 52 PD patients, divided equally between those with and without dyskinesia and 26 healthy controls. Significant differences in the levels of IL-6, TNF-α, visfatin, and progranulin were noted between the groups. Patients with dyskinesia exhibited notably higher IL-6 levels compared to controls, and TNF-α was significantly elevated in both PD patient groups relative to the control group. Additionally, visfatin levels were higher in PD patients without dyskinesia as opposed to those with dyskinesia, and progranulin levels were elevated in the non-dyskinetic PD group compared to controls. The findings highlight the potential role of the examined biomarkers in the pathophysiology of PD. Changes in levels of the tested inflammatory biomarkers and adipokines might be associated with Parkinson’s disease and its symptoms such as dyskinesia.

## 1. Introduction

Parkinson’s disease (PD) is a progressive neurodegenerative disorder that primarily affects the motor system, leading to significant physical and functional impairments [1]. PD, ranked as the second most prevalent neurodegenerative disorder globally, is witnessing a significant rise in incidences [2]. Current projections estimate that the number of individuals affected by this disease could potentially double within the next three decades [2]. The disease typically manifests in individuals over the age of 60, although younger people can also be affected, a condition known as young-onset PD [3]. The hallmark symptoms of PD include resting tremor, bradykinesia (slowness of movement), muscle rigidity, and postural instability [4]. These motor symptoms result primarily from the degeneration of dopamine-producing neurons in the substantia nigra pars compacta (SNc) [5]. However, PD also encompasses a range of non-motor symptoms, such as cognitive impairment, mood disorders, sleep disturbances, and autonomic dysfunction, which can significantly affect the quality of life [6]. The cause of PD is not fully understood, but it is believed to involve a combination of genetic and environmental factors [7]. The pathological hallmark of PD includes the presence of Lewy bodies, abnormal protein aggregates that develop inside nerve cells, affecting their function and leading to cell death [8]. Currently, there is no cure for PD, and treatment is primarily focused on managing symptoms [9]. Pharmacological treatments, such as levodopa, dopamine agonists, and inhibitors of type-B monoamine oxidase (MAO-B), are commonly used to alleviate motor symptoms by increasing dopamine levels or mimicking its action in the brain [10]. In addition to medication, surgical therapies like deep brain stimulation are sometimes used in advanced cases [11]. Non-pharmacological interventions, including physical therapy, occupational therapy, and speech therapy, play a crucial role in managing the disease [12].

Dyskinesias constitutes a group of involuntary and disrupted movements occurring in the course of PD [13]. Chorea constitutes a set of short, uncoordinated movements in the form of sudden contractions and motions [14]. Ballism, less common, is a variant of chorea characterized by large-amplitude flinging movements involving proximal extremities [15]. Dystonia is a condition marked by unintended muscle contractions, featuring slow and repetitive motions or abnormal body positions. These movements have the potential to impact various muscles or muscle groups across the entire body [16].

The use of levodopa, especially in long durations and at high doses, is suspected of causing levodopa-induced dyskinesias (LIDs) [14]. Depending on the time elapsed since the administration of the dose, clinical manifestations can be categorized into three types [17]. Peak-dose dyskinesia (PDD) occurs during the peak concentration of L-dopa (during the so-called ON phase when the medication is active). Biphasic dyskinesia (BD) involves intermediate concentrations of L-dopa, at the beginning and end of its action (also belonging to the ON phase). Additionally, one can distinguish dystonia in the OFF phase (when the medication is at a very low concentration) [18]. PDD most commonly manifests as chorea, ballism, and sometimes dystonia, primarily affecting the upper body. On the other hand, BD primarily involves bilateral lower limbs in the form of dystonia or stereotyped movements [19].

Inflammation is a critical response of the body’s immune system, triggered by various factors such as infection, injury, or exposure to harmful substances. Defense mechanisms are designed to protect against the mentioned threats and initiate the healing process [20]. Neuroinflammation, a specific type of inflammation occurring in the brain and spinal cord, is particularly relevant in the context of neurodegenerative diseases like PD [21]. In PD, neuroinflammation is characterized by the activation of microglia, the brain’s primary immune cells [21]. These cells can adopt different states, playing both protective and harmful roles in PD [22]. On the one hand, microglia contribute to neuronal death by producing inflammatory factors. On the other hand, they can also have protective functions, such as producing neurotrophic factors that support neuron health [22]. Dysfunctional phagocytosis in glial cells due to lysosomal defects caused by PD-related mutations contributes to neuroinflammation [23]. Additionally, extracellular α-synuclein, a protein closely linked with PD pathology, can activate microglia in conformation- and mutation-specific manners [24]. This activation, particularly involving the Nod-like receptor (NLR) family in the pyrin domain-containing 3 (NLRP3) inflammasome signaling pathway in microglia, highlights the nuanced interaction between α-synuclein and the innate immune response in PD [25].

Contaldi et al. [26] investigated the role of peripheral adaptive immunity in PD, specifically focusing on transcription factors in CD4+ T cells to differentiate between PD patients with and without motor complications. Patients without motor complications had higher mRNA levels of the signal transducer and activator of transcription 1 (*STAT1*) and the nuclear receptor 4A2 (*NR4A2*), whereas patients with motor complications had higher mRNA levels of the signal transducer and activator of transcription 6 (*STAT6*). These factors were identified as potential biomarkers to differentiate between PD patients with and without motor complications. Thus, these distinct molecular signatures in CD4+ T cells could serve as biomarkers for motor complication development in PD patients. Typically, in the course of inflammation, a change in the levels of selected biomarkers is observed. C-reactive protein (CRP), interleukin-6 (IL-6), and tumor necrosis factor α (TNF-α) may serve as biomarkers for neuroinflammation, often elevated in conditions like PD [27]. Their levels indicate the severity of inflammatory processes in the brain, aiding in understanding disease progression [27]. Additionally, the abundance of studies on various diseases and the ease of their measurements justifies exploring their potential as markers for the progression of PD [28,29]. A chronic inflammatory state not only exacerbates neurodegeneration but also appears to have a systemic impact, influencing various bodily processes including coagulation. Coagulation disorders in PD patients present another layer of complexity. Research suggests that the chronic inflammation associated with PD can disrupt the balance between clot formation and dissolution, leading to an altered coagulation state [30]. This disruption can increase the risk of developing blood clots or thrombotic events, adding to the disease burden.

Adipokines, including visfatin and progranulin, are increasingly recognized for their role in the neuroinflammatory processes associated with PD [31]. As bioactive molecules primarily secreted by adipose tissue, adipokines have significant implications in various physiological processes, including inflammation [32]. In the context of PD, adipokines contribute to the complex interplay between the systemic metabolic state and neuroinflammation. Understanding the role of adipokines in PD offers valuable insights into the disease’s pathophysiology and potential therapeutic targets, particularly in modulating neuroinflammation and its impact on neuronal health [33].

Vitamin D has emerged as a topic of interest in PD due to its potential influence on neuroinflammation and neuroprotection [34,35]. Research data indicate a correlation between lower levels of vitamin D and the severity of PD, suggesting its involvement in the disease’s progression [34]. The properties of vitamin D in immune regulation and the potential to modulate neuroinflammatory responses, especially in the brain’s immune cells, point to its role in the pathophysiology of PD [36]. This association has sparked interest in exploring vitamin D supplementation as a potential therapeutic approach to mitigate neuroinflammation and possibly slow the progression of PD, although further research is needed to fully understand its impact.

The aim of this study was to compare the levels of certain analytes, including IL-6, TNF-α, CRP, visfatin, progranulin, and 25(OH)-vitamin D, across three distinct groups, namely PD patients without dyskinesia, PD patients with dyskinesia, and healthy controls. The classification of PD patient groups based on the occurrence of dyskinesia should clearly distinguish patients in the early stages of the disease from those who have been diagnosed and undergone treatment for a longer period, indicating a more advanced stage of their condition. Enhanced comprehension of neuroinflammation in PD may lead to therapies that significantly improve the quality of life for patients. The parameters have been selected based on their potential role in the progression of PD, ease of measurement, and the potential to serve as markers, given the aforementioned criteria [21]. It is worth noting that the parameters we selected have not been measured within such groups previously. IL-6 and TNF-α are recognized markers of inflammation. Their use is becoming more and more widespread not only in scientific research but also in clinical practice [37,38]. Furthermore, we enriched our study with measurements of adipokines and vitamin D, which are also suspected to play a role in modulating inflammation. The variations in the examined molecules might be intricately linked to the pathophysiology and symptomatology of PD.

## 2. Materials and Methods

### 2.1. Study Subjects

In this research, an examination was carried out on 52 individuals diagnosed with PD. These individuals were segregated into two categories based on their evaluation results from the Hoehn–Yahr scale [39], the unified Parkinson’s disease rating scale (UPDRS) [40] and the Schwab and England ADL (activities of daily living) scale, a recognized metrics for gauging PD severity and the manifestation of dyskinesia. The first category included 26 patients who did not exhibit dyskinesia, while the second encompassed 26 patients with dyskinesia symptoms. Additionally, a control group comprising 26 healthy volunteers was also scrutinized for comparative analysis. Care was taken to ensure that the groups were comparable in terms of gender, age, and body mass index (BMI). A distinct variation was noted in the time elapsed since PD diagnosis among the patient groups. Comprehensive anthropometric and clinical profiles of the study participants are presented in Table 1. The study protocol received the sanction from the Bioethics Committee of the Nicolaus Copernicus University in Toruń functioning at Collegium Medicum in Bydgoszcz, Poland, under consent reference KB 428/2017. In compliance with ethical standards, written informed consent was obtained from all participants before their inclusion in the study.

Each patient group in the study underwent appropriate pharmacological therapy. The treatment, prescribed by an expert physician for every patient, consisted of a mix of benserazide and levodopa. Benserazide acts as a blocker of extra-cerebral aromatic amino acid decarboxylase, enhancing levodopa’s bioavailability in the central nervous system’s target cells. Table 2 details the therapeutic protocol for PD in the study cohorts. The patients classified in the dyskinesia group presented symptoms typical of PDD. PDD is a result of using high doses of levodopa for a long time.

### 2.2. Study Design

Blood samples for the study were obtained at the “Neuromed” Medical Center, located in Bydgoszcz, Poland. This procedure was carried out by highly qualified medical personnel, ensuring accuracy and consistency. The sampling process involved drawing blood from the median cubital vein of the participants, specifically scheduled between the early hours of 7:00 a.m. and 9:00 a.m. In this study, the inclusion criteria for participants involved those diagnosed with PD, who were then categorized into two distinct groups. A key aspect of the inclusion criteria was the requirement that these patients be free from other diseases, thereby focusing the study exclusively on the impacts of PD. The control group was composed of completely healthy individuals, carefully selected to ensure the absence of any history of neurological disorders or significant chronic conditions. Exclusion criteria were stringently applied to all participants, including both PD patients and control group members. Individuals with any acute or chronic diseases, such as infectious, autoimmune, genetic, inflammatory, or cardiovascular conditions, were excluded. Furthermore, those with a history of other neurological disorders or significant mental health issues that could influence the study’s outcomes were also not included. The selection of participants was conducted through a thorough process involving detailed medical interviews and an analysis of medical records. This approach ensured that the study groups were homogenously constituted in terms of health status, barring the PD condition in the patient groups. Such meticulous selection was pivotal in achieving a clear delineation between the effects of PD and other potential health variables, thereby enabling a more accurate and reliable interpretation of the study’s findings.

### 2.3. Biochemical Analysis

The biochemical analysis was centered on measuring the serum concentrations of several key biomarkers: IL-6, TNF-α, CRP, visfatin, progranulin, and 25(OH)-vitamin D. A variety of commercially available enzyme-linked immunosorbent assay (ELISA) kits were used for these measurements. Specific kits were employed for each biomarker: IL-6 was measured using the Interleukin-6 Human ELISA kit from BioVendor (Brno, Czech Republic), TNF-α with the Human TNF-alpha High Sensitivity ELISA kit from BioVendor (Brno, Czech Republic), CRP using the C-reactive Protein ELISA kit from Immundiagnostik AG (Bensheim, Germany), visfatin with the Human Visfatin (NAMPT) ELISA kit from BioVendor (Brno, Czech Republic), progranulin using the Progranulin Human ELISA kit from BioVendor (Brno, Czech Republic), and 25(OH)-vitamin D with the IDK^®^ 25-OH-Vitamin D ELISA kit from Immundiagnostik AG (Bensheim, Germany). The ELISA method, known for its high sensitivity and specificity, involves the binding of antigens to corresponding antibodies in the kit’s microplate wells. All analyses strictly adhered to the manufacturer’s guidelines. The enzyme immunoassay kits used in the study were equipped with all the necessary reagents for conducting the analysis, including standard concentration analytes, as well as blank and control samples. The concentrations of IL-6 and TNF-α were expressed in picograms per milliliter (pg/mL), while the measurements for visfatin, progranulin, and 25(OH)-vitamin D were reported in nanograms per milliliter (ng/mL). The CRP level was recorded in milligrams per liter (mg/L). In addition, coagulation parameters were determined by a certified hospital medical laboratory, adding an extra dimension of comprehensive analysis to the study.

### 2.4. Statistical Analysis

Statistical analysis in this study was conducted using Statistica 13.3 (TIBCO Software Inc., Palo Alto, CA, USA) and Python 3.8.10 (Python Software Foundation, Wilmington, DE, USA), utilizing the pandas (version 1.4.3), matplotlib (3.1.3), statsmodels (0.13.5), and scipy (version 1.10.1) libraries. The results were represented as means, standard error of the mean (SEM), median, and interquartile range (IQR). To assess the equivalence of the groups, the chi-square test was employed. Normality was examined using the Shapiro–Wilk test, and equality of variances was verified using Levene’s test. In addition to these tests, the Student’s t-test was used for comparing two independent groups, and the chi-square test of independence was applied for analyzing categorical variables. For the main analysis, if the data satisfied the criteria of normal distribution and equal variances, a one-way ANOVA with the Tukey HSD post hoc test was performed. If the data met the normal distribution criterion but not the equal variance criterion, a one-way ANOVA with the T2 Tamhane post hoc test was utilized. When the data did not meet the normal distribution criterion, the Kruskal–Wallis test was conducted, followed by the Mann–Whitney U test with a Bonferroni correction for post hoc analysis. In this study, Spearman’s correlation coefficient was employed to analyze the relationships among the various parameters measured. This multifaceted statistical approach ensured a thorough and accurate evaluation of the study’s data. The level of significance was set at *p* < 0.05.

## 3. Results

**Figure 1:**
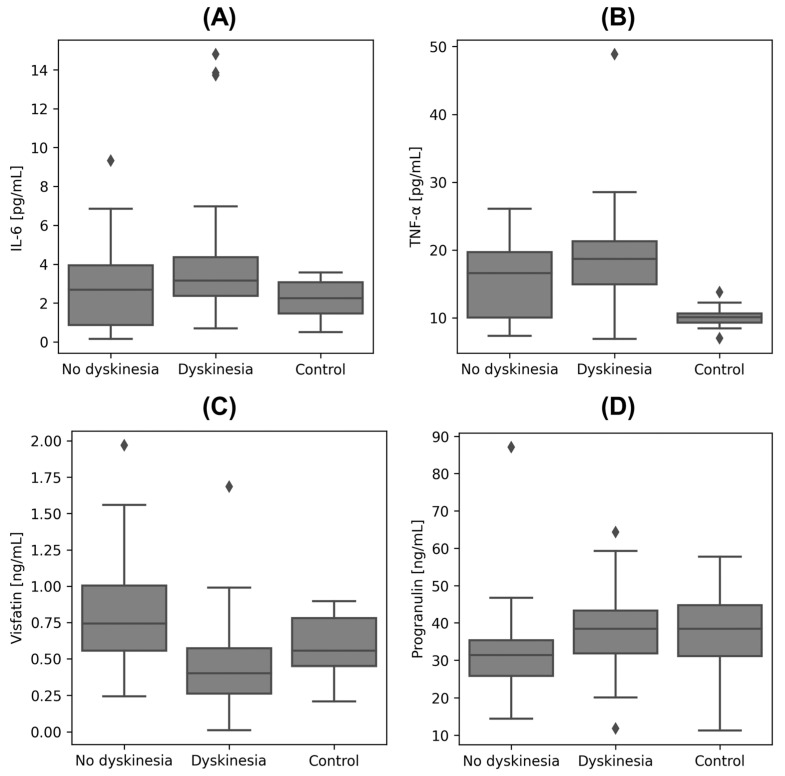
Boxplots for biomarkers that demonstrated statistically significant differences in post hoc analysis. These boxplots include data from two groups of PD patients, as well as the control group. This visual representation provides a clear comparison of the biomarker levels, highlighting the biochemical variances associated with PD. (**A**): boxplot for concentration of interleukin-6 (IL-6); (**B**): boxplot for concentration of tumor necrosis factor α (TNF-α); (**C**): boxplot for concentration of visfatin; (**D**): boxplot for concentration of progranulin.

The laboratory findings presented in Table 3 revealed significant differences between the groups in the concentrations of IL-6, TNF-α, visfatin, and progranulin. The outcomes of the post hoc analysis are detailed in Table 4 and Figure 1. The analysis focused on the differences between groups: non-dyskinesia vs. with dyskinesia, non-dyskinesia vs. control, and with dyskinesia vs. control. The study’s findings showed that, in PD patients with dyskinesia, IL-6 levels were significantly elevated (*p* < 0.05) compared to the controls. TNF-α levels were higher (*p* < 0.0001) in both PD groups when compared to the control group. Visfatin levels increased (*p* < 0.001) in PD patients without dyskinesia versus those with dyskinesia. Additionally, progranulin levels were higher (*p* < 0.05) in the PD patients without dyskinesia compared to the control group.

**Figure 2:**
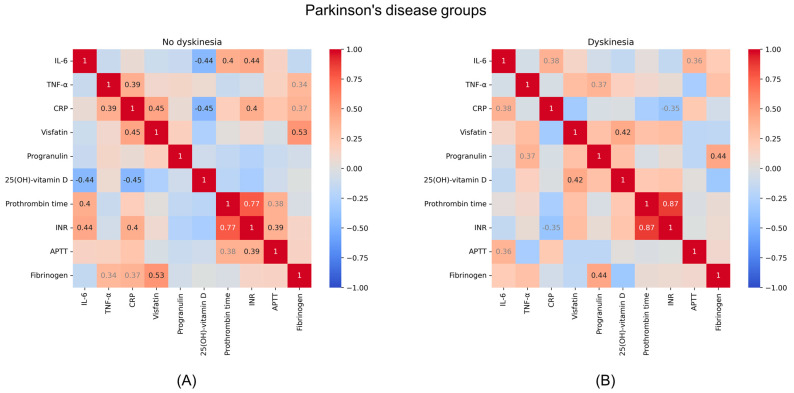
Spearman’s correlation diagrams for different groups of Parkinson’s disease patients. Diagram (**A**) focuses on the group without dyskinesia, while diagram (**B**) pertains to the group with dyskinesia. Abbreviations used: APTT: activated partial thromboplastin time; CRP: C-reactive protein; IL-6: interleukin-6; INR: international normalized ratio; TNF-α: tumor necrosis factor α.

The statistical analysis in this study also encompassed the examination of correlations between various parameters within each group of participants. In the non-dyskinesia group, a significant negative correlation at *p* < 0.05 was found between IL-6 and 25(OH)-vitamin D (R = −0.44). Other notable correlations included CRP with TNF-α (R = 0.39), visfatin (R = 0.45), and 25(OH)-vitamin D (R = −0.45), as well as between visfatin and fibrinogen (R = 0.53). In the dyskinesia group, there were significant correlations between fibrinogen and progranulin (R = 0.44), 25(OH)-vitamin D and visfatin (R = 0.42). Figure 2 shows Spearman’s correlation diagrams for the examined parameters in the PD patient groups.

In order to explore the complex relationships between group classification of patients, specific biomarkers, and other variables, a multiclass logistic regression model was employed. This model primarily focused on predicting the classification of patients into two distinct groups: PD patients without dyskinesia and PD patients with dyskinesia. The predictive variables incorporated into the model included gender and various biomarkers under study. The performance of the regression model was evaluated, yielding a pseudoR^2^ of 0.4293. This, along with an Akaike information criterion (AIC) of 133.8 and a Bayesian information criterion (BIC) of 176.2, suggests a moderate level of predictive quality. Detailed outcomes of the model for each group comparison are presented in Table 5. While comparing the PD patients without dyskinesia with the PD patients with dyskinesia, the results indicated no significant differences in the parameters tested, except for visfatin. Visfatin showed a notable difference (odds ratio (OR) = 1.036, *p* = 0.0076).

## 4. Discussion

PD is a prevalent cause of disability, with a patient’s quality of life being influenced by various factors, including the response to pharmacotherapy and individual disease progression rates. Biochemically, PD is characterized by alterations in the concentrations of pro- and anti-inflammatory factors, underscoring the inflammatory nature of pathogenesis and progression of neurodegeneration. In the present study, the serum level of TNF-α was significantly higher in the PD patients, both with and without dyskinesia compared to the control group, whereas progranulin was increased only in the PD non-dyskinesia patients. Moreover, the serum concentration of IL-6 was significantly elevated in the PD dyskinesia patients versus the control group. Interestingly, the visfatin level was significantly higher in PD patients without dyskinesia when compared with those with dyskinesia. The statistically significant differences between the analyzed groups were not found in the case of CRP or 25(OH)-vitamin D. Our study also delved into the correlations between the analyzed biomarkers within each patient group. In the non-dyskinetic PD group, there were significant correlations between IL-6 and 25(OH)-vitamin D, as well as between CRP, TNF-α, visfatin, and vitamin D. This suggests a nuanced interplay between inflammation, adipokine levels, and vitamin D status in PD. In the dyskinetic PD group, correlations between fibrinogen and progranulin, and vitamin D and visfatin, were observed, highlighting the complex relationships among these markers in PD progression.

IL-6, secreted by monocytes and microglia is associated with neural damage in the course of PD [21]. In addition to promoting the differentiation of activated B cells into immunoglobulin-producing cells, it also regulates processes such as angiogenesis and T cell differentiation [41]. IL-6 is able to inhibit neutrophil accumulation and antagonizes the actions of interleukin-1β (IL-1β) via induction of the receptor antagonist. Furthermore, IL-6 is required to control the levels of pro-inflammatory TNF-α, after endotoxic insults [42]. On the other hand, in the context of the role of IL-6, it is worth mentioning the potentially opposite anti-inflammatory actions of IL-6 in the periphery and in the CNS, which may be explained by the polymorphism of the receptor in different cells [43]. The anti-inflammatory action of IL-6 is mainly through its role in the later stages of inflammation. Once the acute phase of inflammation begins to resolve, IL-6 contributes to the suppression of pro-inflammatory cytokines and the stimulation of anti-inflammatory cytokines [44]. Additionally, IL-6 plays a role in transitioning from innate to adaptive immunity. It can stimulate the growth of IL-4-producing T cells, which are associated with anti-inflammatory responses [45]. Significantly higher serum IL-6 concentrations have been previously reported in PD patients [43,46]. However, the division of the study groups was different than in our study. Interestingly, we found that the IL-6 concentration was over two times higher when comparing dyskinetic patients with the healthy controls. However, we did not demonstrate a statistically significant difference between non-dyskinesia patients and the control group. The increased concentrations of IL-6 in the cerebrospinal fluid (CSF) have been found in PD patients [21,43], but Karpenko et al. [43] showed that it correlated inversely with the Hoehn–Yahr scale score. Moreover, similarly to the presented results, they showed that the non-tremor form of PD had significantly lower serum IL-6 concentrations [43]. Moreover, Bartl et al. [47] demonstrated a positive correlation between the CSF α-synuclein and IL-6. Nevertheless, researchers did not demonstrate correlations of IL-6 in CSF with the development and progression of PD [47]. In the course of PD, IL-6 has been found to correlate with the severity of non-motor symptoms in the form of depression, as well as the rate of death [21]. There is no consensus among researchers on the correlation between IL-6 levels and the rate of PD progression. According to Karpenko et al. [43] the correlation exists, while Simon et al. [48] reject this possibility. The different role of IL-6 during different phases of PD can be attributed to potential polymorphisms in the IL-6 receptor.

In our study, we detected significantly higher TNF-α concentrations in both the dyskinesia and non-dyskinesia PD groups compared to the control group. This observation aligns with previously reported findings, showing higher concentrations of TNF-α in PD patients [49]. It has been reported that TNF-α levels correlated with an increase in the Hoehn–Yahr scale [49], which is not consistent with our study. We found no statistically significant difference in the TNF-α concentration between the PD patients with and without dyskinesia. However, some other studies have found different results [43,50]. Interestingly, significantly higher levels of TNF-α in CSF than in blood serum have been previously reported. Moreover, a positive correlation has been noted between the TNF-α in CSF and the rate of PD progression according to the Hoehn–Yahr scale. Additionally, Karpenko et al. [43] demonstrated a relationship between TNF-α concentration and the PD phenotype, stating that the TNF-α levels in CSF are significantly lower in the group with severe symptoms in the form of tremors. Interestingly, lower serum TNF-α concentrations have been determined in patients with mild cognitive impairment. This picture of changes in TNF-α concentrations may indicate that TNF-α plays an important role in the CNS, even if the TNF-α levels in peripheral blood serum do not reflect this effect [43]. TNF-α can be produced by active monocytes and microglia in the course of neurodegenerative diseases [50]. Increased levels of TNF-α have been noticed directly in the striatum and SNc [51]. TNF-α is neurotoxic and leads to the death of astrocytes, oligodendrocytes, and neurons. This persistent inflammation in the CNS also affects the overall activity of the innate immune system [49]. The expression of TNF-α receptor does not influence the development of PD, but polymorphisms of the TNF-α gene have been detected in PD patients [50,52]. Intriguingly, a significant reduction in TNF-α after the treatment with pramipexole in combination with levodopa has been demonstrated, correlating with the improvement in the UPDRS score [53]. Thus, the changes in TNF-α concentrations might be used as a marker of response to the therapy.

CRP is an acute-phase protein regulated by pro-inflammatory cytokines, used as the common marker of systemic inflammation [54]. Monomeric CRP may increase the permeability of the blood-brain barrier (BBB), allowing pro-inflammatory cytokines to enter the CNS [28]. This process may lead to neuroinflammation and the progression of neurodegeneration. In our study, differences between examined groups did not reach statistical significance. However, there exist contrary reports. Elevated CRP levels have been found in PD patients [55]. Thus, the thesis that the neurodegeneration process, characteristic of PD, is the culprit of elevated CRP levels might be considered [54]. An increase in CRP concentration might be recognized as a prognostic factor for the worsening of both non-motor and motor symptoms in PD patients [56]. Surprisingly, it has been determined that, in PD patients with elevated CRP levels, slower progression of cognitive disorders might be expected [57]. From this perspective, it is worth considering whether inflammation should be reduced in the context of non-motor symptoms. Interestingly, correlations between CRP levels in CSF and the Movement Disorder Society-sponsored revision of the unified Parkinson’s disease rating scale (MDS-UPDRS) III and Montreal Cognitive Assessment (MoCA) scores have been detected [58]. The concentration of CRP in the CSF of women was correlated with the development of non-motor symptoms according to the MoCA, while, in men, CRP in the CSF was correlated with the development of non-motor symptoms according to both scales, MoCA and MDS-UPDRS III.

Visfatin, primarily secreted by adipocytes, is often released concurrently with certain pro-inflammatory cytokines, including TNF-α and interleukins [59]. Visfatin is also known as a pre-B-cell enhancing factor (PBEF) and nicotamide phosphoribosyltransferase (NAMPT) [60]. NAMPT activity is responsible for regulating the levels of NAD^+^ through the synthesis of nicotamide mononucleotide (NMN) [61]. It has endocrine and paracrine functions, influencing glucose metabolism or inflammation regulation [60]. NAMPT is involved in the synthesis of NAD^+^, which in turn regulates the inflammatory response of astrocytes by reacting with the CD38 enzyme [62]. Visfatin has been previously described as a marker for cancer, cardiometabolic, and neurodegenerative diseases [59,60,62]. In vitro studies have shown a decrease in NAMPT expression and NAD^+^ concentrations while exposed to 6-hydroxydopamine (6-OHDA), compared to the control [63]. The extent of this reduction was observed to be proportional to the amount of 6-OHDA introduced. Conversely, the concentration of NADH remained unchanged. It is theorized that 6-OHDA contributes to the death of dopaminergic cells, a process linked to the symptoms and progression of PD [47]. In the presented study, a significantly higher visfatin level in the PD non-dyskinesia patients compared to the PD dyskinesia patients was detected. This level was also higher than in the controls but without reaching a statistically significant difference. This is consistent with the results of other studies indicating a protective role of visfatin in the early stages of PD. Among other things, in studies on PD patients, an increase in the mRNA of the *NAMPT* gene was observed, which may be explained as a mechanism that counteracts oxidative stress and, therefore, the progression of neurodegeneration [64]. In the study by Santiago et al. [64], such a correlation was demonstrated in a group of patients in the early phase of PD, not yet treated with drugs, compared to healthy controls. This may be explained by the efficiency of neuroprotective mechanisms through the action of NAMPT in the early stage of progressive neurodegeneration. The increased amount of the *NAMPT* gene mRNA was postulated as a potential biomarker for PD, while visfatin itself may be used in combating the neurotoxicity of 6-OHDA in the sense of a therapeutic method [64].

Progranulin is a protein with anti-inflammatory and neurotrophic effects [65,66]. Progranulin is considered as a factor supporting the survival of neurons, among other things, by modulating neuroinflammation and by influencing neuronal lysosomes and autophagosomes [67,68]. Mutations in the gene coding for progranulin may lead to frontotemporal dementia, while the deficiency of progranulin itself may contribute to an increase in the concentration of α-synuclein, which may be directly associated with PD [69]. Paradoxically, progranulin may be secreted by activated microglia. The level of progranulin produced by microglia is increased in response to anti-inflammatory cytokines. In contrast, astrocytes show an inverse response, with their progranulin levels increasing due to pro-inflammatory cytokines [70]. In our study, we found that progranulin concentrations were significantly lower in PD patients without dyskinesia compared to the controls. This result is partly consistent with other studies. It has been previously reported that the concentration of progranulin was negatively correlated with an increase in the Hoehn–Yahr scale [67,71]. However, we did not detect any statistically significant differences in progranulin concentrations between the PD groups without and with dyskinesia [67,68,69,70]. Extracellularly, progranulin acts through the ephrin type-A receptor 2 (EphA2), exerting activities as an axonal growth factor and a neuronal survival factor. In the context of Parkinson’s Disease (PD), two loci, namely rs2269906 and rs850738, have been identified as risk variants for the disease [72].

Vitamin D, comprising cholecalciferol (vitamin D3) and ergocalciferol (vitamin D2), plays a crucial role in calcium and phosphorus metabolism, impacting bone health [73]. Obtained from dietary sources and synthesized under ultraviolet B (UVB) exposure, both forms are converted to calcidiol 25(OH)D in the liver, and then, in the kidney, to the active metabolite calcitriol—1,25(OH)_2_D [74]. Beyond bone health, vitamin D has been found to perform various physiological functions, including modulation of immune responses. The comprehensive meta-analysis conducted by Mousa et al. [75] offers robust level 1 evidence, indicating that vitamin D supplementation can be effective in reducing chronic low-grade inflammation among individuals with type 2 diabetes. The 1,25(OH)_2_D is pivotal in the regulation of immune responses within the brain. It acts on microglia and astrocytes, the resident immune cells of the CNS, and inhibits the production of pro-inflammatory cytokines, such as IL-6 and TNF-α [76,77,78]. This inhibition is achieved through the blockade of pathways like nuclear factor kappa-light-chain-enhancer of activated B cells (NF-κB), a key regulator of inflammation [76]. Neuroinflammation, often a result of reactive oxygen species (ROS), nitric oxide, and cytokine overproduction by activated glia, leads to neuronal damage. Thus, low levels of vitamin D3 might be associated with various brain diseases. In the study by Huang et al. [77], researchers explored the role of 1,25(OH)_2_D in mitigating neuroinflammation induced by lipopolysaccharides (LPSs) in primary cortical neuron-glia cultures. They found that LPSs stimulated the production of nitrite, ROS, IL-6, and macrophage inflammatory protein (MIP)-2. Inhibition of specific mitogen-activated protein kinase (MAPK) pathways reduced the levels of these neuroinflammatory markers. Notably, 1,25(OH)_2_D effectively attenuated the LPS-induced production of these inflammatory molecules and inhibited the phosphorylation of MAPK pathways. The results suggest that 1,25(OH)_2_D may have a therapeutic potential in reducing neuroinflammation in brain injuries. According to the analysis by Pignolo et al. [35], there is emerging evidence that low levels of serum 25(OH)-vitamin D may be linked to an increased risk of PD onset, while higher levels appear to correlate with improved motor functions, particularly in balance control. The effectiveness of vitamin D supplementation as a complementary approach to pharmacological and rehabilitative therapy in PD patients has not been conclusively established. In our study, we did not observe statistically significant differences in the levels of serum 25(OH)-vitamin D between the study groups. It is worth emphasizing that the concentrations of 25(OH)-vitamin D serum determined in the presented study are below the optimal range for humans for all study groups. Following the guidelines by Płudowski et al. [79], deficiency in total serum 25(OH)-vitamin D is indicated by levels below 20 ng/mL. Concentrations between 20 and 30 ng/mL are considered suboptimal, while optimal vitamin D levels fall within the range of 30–50 ng/mL. Vitamin D deficiency is a widespread issue across both developing and developed countries, particularly in regions with limited exposure to solar radiation due to their geographical location. This is notably evident in countries like Poland, where vitamin D deficiencies are commonly observed due to its specific geographical and climatic conditions [80,81,82]. However, the connection between vitamin D levels and the severity or clinical progression of PD symptoms remains unclear, leaving its potential as a biomarker for disease progression uncertain. More research is needed to clarify vitamin D’s role in the etiology of PD, its impact on both motor and non-motor symptoms, quality of life, and disease progression.

**Figure 3:**
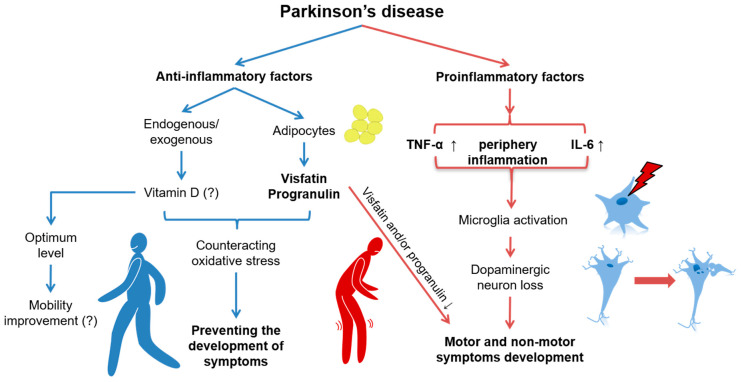
Putative relationship between inflammation and Parkinson’s disease. Decline in anti-inflammatory agents, both internally produced and externally derived, is expressed by reduced secretion of visfatin and progranulin by adipocytes, alongside a tentative association with vitamin D status. Conversely, there is an escalation in proinflammatory agents that activate microglia, which could be related to periphery inflammation with increased levels of tumor necrosis factor α (TNF-α) and interleukin-6 (IL-6) in the blood plasma. These changes can be connected with the pathogenesis of Parkinson’s disease.

Figure 3 outlines the dynamic interplay between the inflammatory processes in the context of PD. On one side, anti-inflammatory factors, both endogenous and exogenous, are shown to decrease, which is highlighted by the reduction in adipocyte-secreted visfatin and progranulin, as well as an uncertain relationship with vitamin D levels. On the other side, an increase in proinflammatory factors leads to microglia activation, resulting in elevated levels of TNF-α and IL-6.

The primary limitation of this preliminary study is identified as the relatively small sample size of participants. Consequently, the findings may not accurately represent the broader population. This small sample size resulted from the stringent inclusion and exclusion criteria implemented to ensure the homogeneity of the study groups. While beneficial for controlling variables, such criteria inevitably lead to a reduced pool of participants.

In studies with limited sample sizes, like this one, ensuring that the sample genuinely represents the target population becomes a significant challenge. This can introduce biases, potentially skewing the results. Furthermore, the statistical power of this study may be impacted by the small number of participants, which could hinder the detection of significant differences between groups. Despite these challenges, it is noteworthy that comprehensive and advanced statistical analyses were conducted in this study. These analyses successfully identified statistically significant differences, even with the limited number of participants. However, the results should be interpreted with caution, and further research with a larger sample size is recommended to validate and expand upon these findings. The different duration of the disease in the patients from the groups without and with dyskinesia may be a potential limitation of the study. Titova et al. [83] indicate that the duration of PD is not a reliable measure for assessing the severity of the condition. Instead, specific symptoms exhibited by a patient and the stage of progression of the disease are more critical factors to consider. However, in the presented study, the different stages of the disease in both groups were confirmed with specific scales used to assess the severity of PD. Undoubtedly, there is a need for a more nuanced understanding of PD, with particular emphasis on not only the time since diagnosis but also the nature and intensity of symptoms and the stage of disease advancement. This preliminary study lays the groundwork, offering insights and directions for future research in this area.

## 5. Conclusions

The results of this presented study provide valuable insights into the inflammatory and metabolic changes associated with PD and its symptoms, particularly dyskinesia. They pave the way for future research to explore the role of the analyzed biomarkers in PD more deeply, potentially leading to novel therapeutic approaches or biomarkers for disease progression. While our study offers a foundational understanding, further research with larger cohorts is essential to validate these findings and explore their implications in the broader context of PD management and treatment.

## Figures and Tables

**Table 1:** Characteristics of patients with Parkinson’s disease (PD) and healthy individuals. A *p*-value of less than 0.05 was considered statistically significant.

Parameter		Parkinson’s Disease		Control	*p*-Value	Power of a Test
		Non-Dyskinesia	With Dyskinesia			
n (Female/Male)		26 (13/13)	26 (12/14)	26 (13/13)	-	-
Age [years]	Mean	67.462	68.423	66.308	0.0933	0.9998
	SEM	1.137	1.154	1.109		
	Median	68.500	68.500	66.000		
	IQR	7.750	6.000	4.750		
Body Mass [kg]	Mean	74.654	73.346	70.308	0.2785	1.0000
	SEM	2.250	2.974	1.897		
	Median	74.500	73.000	86.000		
	IQR	14.750	13.000	16.000		
Height [cm]	Mean	168.692	166.808	167.231	0.5200	0.4212
	SEM	1.542	1.299	0.618		
	Median	168.000	167.500	167.000		
	IQR	11.000	7.500	3.500		
BMI [kg/m^2^]	Mean	26.158	26.221	25.121	0.5025	0.4376
	SEM	0.611	0.941	0.633		
	Median	25.619	25.457	24.562		
	IQR	4.163	3.709	5.346		
SBP [mmHg]	Mean	125.038	125.962	123.423	0.7033	1.0000
	SEM	1.133	2.481	1.702		
	Median	122.500	125.000	123.500		
	IQR	10.000	10.000	14.750		
DBP [mmHg]	Mean	76.923	75.385	73.962	0.8623	1.0000
	SEM	3.972	1.817	1.088		
	Median	72.500	72.500	72.500		
	IQR	10.000	10.000	8.750		
UPDRS I	Mean	0.885	2.346	-	<0.0001	0.9909
	SEM	0.150	0.248	-		
	Median	1.000	2.000	-		
	IQR	1.000	1.000	-		
	Min	0.000	0.000	-		
	Max	2.000	5.000	-		
UPDRS II	Mean	6.154	12.462	-	<0.0001	1.0000
	SEM	0.760	0.728	-		
	Median	6.000	12.000	-		
	IQR	3.000	4.000	-		
	Min	1.000	5.000	-		
	Max	15.000	20.000	-		
UPDRS III	Mean	20.346	38.038	-	<0.0001	1.0000
	SEM	1.331	1.442	-		
	Median	19.000	37.000	-		
	IQR	11.000	7.000	-		
	Min	10.000	26.000	-		
	Max	33.000	54.000	-		
UPDRS IV	Mean	0.077	2.962	-	<0.0001	1.0000
	SEM	0.053	0.218	-		
	Median	0.000	3.000	-		
	IQR	0.000	2.000	-		
	Min	0.000	1.000	-		
	Max	1.000	6.000	-		
Hoehn–Yahr Scale	Mean	1.308	2.904	-	<0.0001	0.9956
	SEM	0.049	0.079	-		
	Median	1.500	3.000	-		
	IQR	0.500	0.500	-		
	Min	1.000	2.500	-		
	Max	1.500	4.000	-		
Schwab and England Activities of Daily Living scale	Mean	87.308	62.308	-	<0.0001	1.0000
	SEM	1.046	1.393	-		
	Median	90.000	60.000	-		
	IQR	10.000	10.000	-		
	Min	80.000	50.000	-		
	Max	100.000	80.000	-		
Years since Diagnosis [years]	Mean	1.615	8.346	-	<0.0001	1.0000
	SEM	0.097	0.271	-		
	Median	2.000	8.000	-		
	IQR	1.000	3.000	-		
	Min	1.000	6.000	-		
	Max	2.000	10.000	-		
Place of Residence [n]	City over 100,000	14	17	12	-	-
	City up to 100,000	5	3	4		
	City up to 50,000	2	4	8		
	Village	5	2	2		
Education [n]	Higher	1	5	13	-	-
	Secondary	14	13	9		
	Vacational	10	7	4		
	Primary	1	1	0		

Abbreviations used: BMI: body mass index; DBP: diastolic blood pressure; IQR: interquartile range; Max: maximum value; Min: minimum value; UPDRS: unified Parkinson’s disease rating scale; SBP: systolic blood pressure; SEM: standard error of mean.

**Table 2:** Pharmacological treatment in analyzed groups of Parkinson’s disease (PD) patients.

Drugs, Dose	PD Non-Dyskinesia [n]	PD With Dyskinesia [n]
no treatment	1	0
madopar HBS (LEDD = 125)	19	12
madopar 250 + HBS (LEDD = 375)	0	7
madopar HBS + madopar 125 (LEDD = 250)	0	4
madopar HBS + madopar 125 + madopar 62.5 (LEDD = 313)	1	1
madopar 62.5 + HBS (LEDD = 188)	4	1
madopar 125 + madopar 125 (LEDD = 250)	1	0
madopar × 4 (LEDD = 500)	0	1

Active ingredients in medicines: madopar HBS: 100 mg levodopa + 25 mg benserazide; madopar 250 + HBS: 200 mg levodopa + 50 mg benserazide; madopar 125: 100 mg levodopa + 25 mg benserazide; madopar 125: 100 mg levodopa + 25 mg benserazide; madopar 62.5: 50 mg levodopa + 12.5 mg benserazide. The acronym HBS stands for a hydrodynamically balanced system. Abbreviations used: LEDD: levodopa equivalent daily dose.

**Table 3:** Comparative analysis of biochemical parameters between patients diagnosed with Parkinson’s disease and healthy individuals serving as a control group. A *p*-value less than 0.05 is regarded as statistically significant.

Parameter		Parkinson’s Disease		Control	*p*-Value	Power of a Test
		Non-Dyskinesia	With Dyskinesia			
IL-6 [pg/mL]	Mean	2.823	4.468	2.180	0.0206	0.9999
	SEM	0.447	0.753	0.188		
	Median	2.685	3.153	2.258		
	IQR	3.054	1.977	1.613		
TNF-α [pg/mL]	Mean	15.879	19.154	10.149	<0.0001	1.0000
	SEM	1.151	1.498	0.272		
	Median	16.606	18.698	10.107		
	IQR	9.605	6.341	1.319		
CRP [mg/mL]	Mean	2.948	3.057	2.340	0.9468	0.6410
	SEM	0.540	0.523	0.164		
	Median	2.300	1.855	2.214		
	IQR	2.620	1.850	1.201		
Visfatin [ng/mL]	Mean	0.818	0.477	0.585	0.0009	0.1751
	SEM	0.080	0.067	0.043		
	Median	0.742	0.402	0.556		
	IQR	0.449	0.310	0.330		
Progranulin [ng/mL]	Mean	32.641	38.141	38.840	0.0215	1.0000
	SEM	2.630	2.360	2.260		
	Median	31.406	38.417	38.421		
	IQR	9.410	11.480	13.686		
25(OH)-vitamin D [ng/mL]	Mean	23.792	22.973	27.917	0.1966	1.0000
	SEM	0.918	1.117	1.808		
	Median	24.050	23.500	27.920		
	IQR	6.725	7.725	14.108		
PT [s] (normal range: 11–13.5 s)	Mean	12.373	11.804	-	0.3780	0.4898
	SEM	0.610	0.155	-		
	Median	11.950	11.600	-		
	IQR	0.550	0.675	-		
INR (normal range: 0.8–1.1)	Mean	1.095	1.037	-	0.2870	0.0546
	SEM	0.058	0.015	-		
	Median	1.050	1.020	-		
	IQR	0.045	0.075	-		
APTT [s] (normal range: 27–41 s)	Mean	30.458	28.746	-	0.1810	0.9976
	SEM	0.927	0.466	-		
	Median	29.450	28.000	-		
	IQR	4.600	2.650	-		
Fibrinogen [g/L] (normal range: 2–4 g/L)	Mean	3.373	3.412	-	0.8020	0.0570
	SEM	0.093	0.121	-		
	Median	3.300	3.300	-		
	IQR	0.675	0.975	-		

Abbreviations used: APTT: activated partial thromboplastin time; CRP: C-reactive protein; IL-6: interleukin-6; INR: international normalized ratio, calculated as (PT patient/PT normal); IQR: interquartile range; PT: prothrombin time; SEM: standard error of mean; TNF-α: tumor necrosis factor α.

**Table 4:** Results of post hoc analysis, with statistical significance determined at *p* < 0.05.

Parameter	*p*-Value		
	Non-Dyskinesia vs. With Dyskinesia	Non-Dyskinesia vs. Control	With Dyskinesia vs. Control
IL-6	>0.05	>0.05	0.012
TNF-α	>0.05	0.003	<0.001
Visfatin	0.002	>0.05	>0.05
Progranulin	>0.05	0.032	>0.05

Abbreviations used: IL-6: high sensitivity interleukin-6; TNF-α: tumor necrosis factor α.

**Table 5:** Comprehensive overview of the odds ratios and their statistical significance for each parameter when comparing Parkinson’s disease patients without dyskinesia to those with dyskinesia.

Parameter	OR [95% CI]	*p*-Value
Sex	2.114; [1.197; 22.589]	0.6905
IL-6	3.229; [2.368; 4.925]	0.3111
TNF-α	2.968; [2.611; 3.431]	0.1877
CRP	2.994; [2.257; 4.38]	0.5444
Visfatin	1.036; [1.003; 1.511]	0.0076
Progranulin	2.826; [2.644; 3.035]	0.2600
25(OH)-vitamin D	2.782; [2.472; 3.181]	0.7132

Abbreviations used: CI: confidence interval; CRP: C-reactive protein; IL-6: interleukin-6; OR: odds ratio; TNF-α: tumor necrosis factor α.

## Data Availability

The data presented in this study are available on request from the corresponding author. The data are not publicly available due to privacy/ethical restrictions.
